# Microsatellites as Molecular Markers with Applications in Exploitation and Conservation of Aquatic Animal Populations

**DOI:** 10.3390/genes14040808

**Published:** 2023-03-27

**Authors:** Roman Wenne

**Affiliations:** Institute of Oceanology, Polish Academy of Sciences, Powstańców Warszawy 55, 81-712 Sopot, Poland; rwenne@iopan.gda.pl

**Keywords:** biodiversity, conservation genetics, aquaculture, molecular population genetics, microsatellite DNA, SNP, allozymes and mtDNA, selection, aquatic organisms

## Abstract

A large number of species and taxa have been studied for genetic polymorphism. Microsatellites have been known as hypervariable neutral molecular markers with the highest resolution power in comparison with any other markers. However, the discovery of a new type of molecular marker—single nucleotide polymorphism (SNP) has put the existing applications of microsatellites to the test. To ensure good resolution power in studies of populations and individuals, a number of microsatellite loci from 14 to 20 was often used, which corresponds to about 200 independent alleles. Recently, these numbers have tended to be increased by the application of genomic sequencing of expressed sequence tags (ESTs) and the choice of the most informative loci for genotyping depends on the aims of research. Examples of successful applications of microsatellite molecular markers in aquaculture, fisheries, and conservation genetics in comparison to SNPs are summarized in this review. Microsatellites can be considered superior markers in such topics as kinship and parentage analysis in cultured and natural populations, the assessment of gynogenesis, androgenesis and ploidization. Microsatellites can be coupled with SNPs for mapping QTL. Microsatellites will continue to be used in research of genetic diversity in cultured stocks, and also in natural populations as an economically advantageous genotyping technique.

## 1. Introduction

The global production of aquatic animals for consumption was almost 178 million tonnes in 2020, including capture fisheries of 90.3 million, and aquaculture production of 87.5 million tonnes [1]. One of the possibilities for further increasing production that has been used in recent decades is the application of scientific research in the fields of biotechnology and genetics [2]. The development of genetics has enabled the discovery of molecular markers suitable for the study of a large number of individuals in natural and breeding populations. A few types of molecular markers used in research associated with aquaculture and fisheries have been described over the last few decades decades [3,4,5,6,7]. Their applications in uncovering the differentiation of natural populations for the purposes of improved fishery management, population genetic structures and local adaptations for conservation biology purposes, identification of selected lines in aquaculture, analysis of kinship in the stocks and identification of individuals, finding quantitative trait loci (QTL) for the purposes of aquaculture, identification of taxa, food products, and forensics have been reported in a large number of papers [8,9,10,11,12,13,14,15,16,17,18,19,20,21,22].

Proteins and allozymes have been continuously used in analyses of natural and hatchery populations since the 1960s and have provided a vast amount of information about their taxonomy, hybridization, genetic polymorphism and spatial differentiation in relation to aquatic environments (Table 1). 

Allozymes were analyzed under assumptions of models based on neutrality, whereas more recently, they have been considered molecular markers operating under natural selection [74,75,76]. Differences between alleles of allozyme loci have been classified as amino acid variants caused by nucleotide substitutions, which often reveal less polymorphisms in comparison to DNA markers such as single nucleotide polymorphisms (SNPs) [77]. The resolution power in population genetic analyses usually confirms a lower diagnostic level of allozymes in finding differentiation compared with mtDNA and microsatellites [78]. Allozymes are rarely but still used successfully as markers for identification of taxa and populations of aquatic organisms, and improvement of hatchery stocks [65,79,80,81].

Mitochondrial DNA (mtDNA) as a molecular marker differs from nuclear DNA due to such properties as maternal inheritance in most animal species and a higher mutation rate due to relaxed selection [82]. A few exceptions, mainly in marine and freshwater mussels, include biparental and doubly uniparental inheritance and very rarely recombination [83,84,85,86,87,88]. MtDNA has been used in studies related to fisheries, aquaculture, conservation genetics, estimation of introgression from restocking, introductions and invasions, phylogenetic and phylogeographic analyses, and seafood testing and analysis (Table 2). 

The marker MtDNA cytochrome c oxidase 1 (COI) has been commonly used for aquatic species identification—barcoding [149,150,151]. However, mtDNA introgression from one species to another has been reported in populations in which hybridization occurs [152,153,154,155]. In studies of population differentiation, mtDNA often shows incongruent results in comparison to allozymes and nuclear DNA markers, which limits its applications as a versatile molecular marker. Nevertheless, mtDNA variation in population studies can be used as one of the tested markers or to solve specific questions concerning the identification of taxa and female line genealogy. 

Nuclear DNA markers have increasingly been used since the 1990s. They were originally based on the detection of single changes in the genome: point mutations or rearrangements in a very limited number of genome sites recognized by cuts with restriction enzymes. DNA marker technologies and their applications in aquaculture genetics have been reviewed several times [82,156,157,158]. Popular genetic markers used in aquaculture-related research include restriction fragment length polymorphism (RFLP), randomly amplified polymorphic DNA (RAPD), amplified fragment length polymorphism (AFLP), simple sequence repeats (SSRs—microsatellites), single nucleotide polymorphism (SNP), and expressed sequence tags (ESTs). The nuclear DNA markers have enabled progress in the assessment of genetic variability and inbreeding, parentage assignments, species and strain identification, and the construction of high-resolution genetic linkage maps for aquaculture species; AFLP can be source of irreproducible results leading to uncertain conclusions, and their use is not recommended for routine population genetics assays. RAPD markers are dominant and do not distinguish between homozygotes and heterozygotes. However, AmpFLP coupled with sequencing is an efficient way of obtaining reliable information about genetic relationships between studied populations. rDNA-ITS markers have been commonly used for species identification–barcoding [159,160,161,162]. Restriction-site associated DNA sequencing (RAD-Seq) and its latest variants coupled with NGS has been extensively applied to generate population-level SNP genotype data [163]. The number of SNPs discovered through the application of high-throughput sequencing for a chosen non-model species can be large (exceeding hundreds of thousands). SNP arrays have been designed for genotyping such a large number of loci [164,165,166,167,168]. However, in studies of populations, a subset of diagnostic SNPs can be employed for cost-effective genotyping in individual laboratories. SNPs and microsatellites are regarded as high-resolution molecular markers. Comparisons of the results of SNP-based and microsatellite-based population studies has often demonstrated the higher accuracy of SNPs. Nevertheless, microsatellites are also effective in the discovery of population processes and kinship analysis and are considered relevant markers in population genetic research [14,169,170,171].

**Figure 1:**
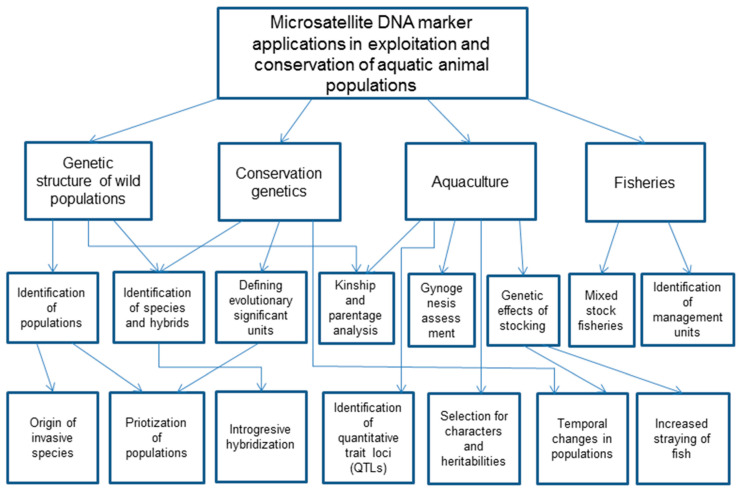
An integrated concept map showing the main fields of applications of microsatellite DNA markers related to aquatic exploited animal populations.

Microsatellites are highly polymorphic, neutral and co-dominant DNA markers based on a variable numbers of short, usually 2–4 bp, nucleotide repeats [172]. Microsatellites have been most widely applied in research related to populations, fisheries and aquaculture beginning in the early 1990s [173,174,175,176]. The next generation sequencing (NGS) genome assay enables the identification of many thousands of microsatellites of which a dozen or so highly polymorphic loci are usually sufficient for population genetic and aquaculture applications, e.g., in [177]. This review is an attempt to summarize the applications of microsatellite DNA markers to studies of natural populations, aquaculture stocks, fisheries and conservation genetics of aquatic animals (Figure 1). 

## 2. Genetic Structure of Wild Populations

Microsatellites have been most useful in terms of genetic characterisations and in the structure analysis of natural populations of many species. Examples of aquatic species for which population genetic structure has been successfully identified are presented in Table 3. 

The list includes freshwater, diadromous and marine species. For the study of the genetic structure of the European hake, Pita et al. [177] distinguished several groups of microsatellite markers. The most commonly used sets of microsatellites are neutral. Microsatellites detected in ESTs are characterized by high allelic diversity in regions of functional importance. This group of microsatellites includes neutral markers with non-functional (non-adaptive) polymorphisms, purified (fossil-adaptive) polymorphism, and stabilizing (adaptive) polymorphism located in the adaptive regions of the genome [177]. In the latter group of markers, outliers can occur. These types of microsatellite markers give different pictures of the genetic diversity of populations, as in the case of the European hake. However, for some marine species, no genetic structuring has been observed. Genetic differentiation has not been found with microsatellites in some migrating oceanic species, such as the squid species *Loligo reynaudii* and *Doryteuthis (Amerigo) pealeii* [213]. Three different marker types, mitochondrial DNA, microsatellites and SNPs, were used in the analysis of 85 archaeological herring bones in an attempt to reconstruct the genetic diversity and population structure of ancient Pacific herring (*Clupea pallasi*) populations from the west coast of North America [214]. MtDNA revealed high haplotypic diversity, which is also present in contemporary populations, but no differences between populations. Microsatellite DNA data quality of ancient samples was very poor due to high allele drop-out and stuttering. SNP data had low error rates and were suitable for finding genetic differentiation. However, a recent study of the tiger shark, *Galeocerdo cuvier*, with microsatellites revealed differentiation between populations from the Atlantic and Indo-Pacific including contemporary and archival samples [215]. 

A simulation study of the accuracy of assigning individuals to closely related populations of chum salmon, *O. keta*, with 15 microsatellite loci exhibiting 349 independent alleles compared with 61 SNPs exhibiting 66 independent alleles, revealed that the SNP baseline performed considerably better than the microsatellite baseline [216]. An equivalent level of individual assignment to populations of chinook salmon, *O. tshawytscha*, in 60 populations from British Columbia obtained with 16 highly polymorphic microsatellite loci was projected to require 179 SNPs [217]. A population-specific estimation for this same species in the Yukon River showed that the nine-SNP baseline was approximately equivalent to a single microsatellite locus with 17–22 alleles [218]. Microsatellites with more alleles provided more accurate estimates of stock composition than those with fewer alleles. Microsatellite DNA markers as neutral markers do not reveal all occurring differences between natural populations, in which adaptive genetic markers, e.g., three circadian clock genes (OtsClock1b, OmyFbxw11, and Omy1009UW) discriminate temporally divergent migratory runs of Chinook salmon in the Feather River [219]. The resolution powers of two sets of SNPs-, a RAD-seq generated SNP panel and SNP (5568 loci) array developed by the Centre for Integrative Genetics (CIGENE, Norway), and a microsatellite panel for the assessment of differences among transatlantic populations of Atlantic salmon, *S. salar*, have been compared [220]. Both SNP sets and the microsatellite panel confirmed genetic divergence between the east and west Atlantic populations. Evidence consistent with introgression among the east and west Atlantic groups was found in the SNP data sets but not in the microsatellite data. That work highlighted the usefulness of multiple marker comparisons in identifying introgression. However, the costs of genotyping a large number of SNPs in populations exceed those of microsatellites. The application of high-throughput sequencing facilitates the discovery and further use of microsatellite markers in studies of genetic structures of wild populations, e.g., [221]. 

## 3. Population Genetics of Invasive Species

Invasions of single or groups of aquatic species have been caused by natural events or human-mediated activities, such as shipping, aquaculture, recreational activities or attempts to enrich local ecosystems, including increasing fishing catches. Despite the understanding of the negative consequences of uncontrolled introductions of species into new areas, their numbers continue to increase, and the economic impact is increasing. There are examples of banning the entry of large ships with hulls contaminated by sessile exotic marine organisms to certain countries, e.g., New Zealand. The use of molecular markers makes it possible to identify invasive species, reconstruct their migration routes and source populations, reveal their hybridization with local congeners, estimate the economic and ecological impacts, predict their further spread and facilitate preventive actions. 

Population genetics of invasive species have been examined by studies of microsatellites in many species, including the evolution of brown trout populations originating from Poland and introduced to virgin rivers systems of the subantarctic Kerguelen Islands [222], smallmouth bass, *Micropterus dolomieu*, introduced to the range of Guadalupe bass, *M. treculii* in central Texas and its introgressive hybridization in a few rivers [223], Asian black carp, *M. piceus*, imported to U.S. aquaculture farms in the 1980 and present in the Mississippi River basin since the early 1990s [224], bighead carp, *Hypophthalmichthys molitrix*, and silver carp, *H. nobilis*, in Hungary, imported from China [225], and signal crayfish, *Pacifastacus leniusculus*, introduced to Europe from North American lakes for hatchery purposes [226]. 

Microsatellites have extended the knowledge of invasive populations of estuarine and marine species, such as rainbow trout, *O. mykiss*, native to the Pacific coast of North America and brown trout, *S. trutta*, from Europe, both introduced from Germany to South America over 100 years ago [227]. They have revealed the successful introduction of sockeye salmon, *O. nerka*, into a new environment, Frazer Lake, Kodiak Island, Alaska [228], estuarine fishes, dusky flathead, *Platycephalus fuscus*, and sand whiting, *Sillago ciliata*, objects of recreational and commercial fishers on the east coast of Australia [229], and the origin (source population) of invasive American brine shrimp, *Artemia franciscana*, in the Mediterranean Sea region [230], Manila clam, *Ruditapes philippinarum*, in the Mediterranean introduced for aquaculture purposes from the Indo-Pacific in 1983 [231,232], invasive brown mussel, *Perna perna*, in the Gulf of Mexico [233], and American Pacific oyster, *C. gigas*, introduced for aquaculture and present as a wild populations in Ireland, France, The Netherlands [234,235] and many other countries. 

## 4. Conservation Genetics

Despite the great understanding of the need to protect entire aquatic ecosystems, actions to protect and restore individual species often remain difficult. One way to support these activities is to plan and manage endangered populations, taking into account their genetic characteristics. Based on the results of population genetics research, it is possible to define evolutionary conservation units [236]. Significant evolutionary units that are communities of individuals of the same species that are adapted to local conditions and reproductively self-sustaining. Supportive breeding and restocking is one way to prevent the decline of populations of aquatic animals [237]. 

Microsatellites have become a tool in the increasing the development and application of conservation genetics, and a few examples of its uses can be listed: analysis of populations of endemic California Paiute cutthroat trout, *O. clarki seleniris*, threatened by hybridization with introduced rainbow trout, *O. mykiss* [12], maintenance of the genetic diversity of steelhead, *O. mykiss*, tributary populations from the Bulkley-Morice River, British Columbia [238], analysis of the genetic variation of rainbow trout, *O. mykiss*, below and above natural barriers and man-made dams in rivers in California [239], description of population subdivision and conservation implications of westslope cutthroat trout (*O. clarki lewisi*) on the northern periphery of its range [240], and the effects of stocking in populations of Chinook salmon, *O. tshawytscha*, in the North Fork Stillaguamish River, WA, USA [241]. Significant genetic differences between seasonal runs of sockeye salmon revealed by microsatellite DNA analysis provide support for the management strategy that has been employed for nearly 20 years to protect the genetic diversity of this species in Bear Lake, Alaska [242]. Microsatellites were used for analysis of native white-spotted charr populations of *Salvelinus leucomaenis* threatened by hybridization with non-native brook trout, *S. fontinalis*, in Japan [243] and analysis of genetic structures among stocked and native populations of the European grayling, *Thymallus thymallus*, in Europe [244]. 

The Fraser River system consisted of five white sturgeon (*Acipenser transmontanus*) management units, two of which were listed as endangered populations under Canada’s Species at Risk Act, which were verified with microsatellite markers [245]. Microsatellites were applied in the assessment of using barriers for the conservation of native salmonid populations threatened by non-native migratory fish invasions [246], analysis of population genetic structure of endangered freshwater pearl mussel (*Margaritifera margaritifera* L.) in Europe, elaboration of the recommendation concerning conservation units [247] and study of population genetics of the exploited oyster, *Crassostrea rhizophorae*, in Brazil [248]. 

In order to monitor spawning success in earthen ponds, individual broodfish of the channel catfish, *Ictalurus punctatus* were identified prior to stocking, by genotype analysis with polymorphic microsatellite DNA markers [249]. Effective population size and demographic rate estimation can be performed with microsatellites, e.g., in wild Atlantic sturgeon, *Acipenser oxyrinchus oxyrinchus* [250]. Translocations and reintroductions have been used to prevent the extinction of freshwater fish populations of the hardyhead, *C. fluviatilis*, in The Murray–Darling Basin in south-eastern Australia [203]. The guided artificial gene flow strategy was based on genetic analyses of 14 microsatellite loci and enabled the rescue of this species from extinction. All 46 species of seahorses, *Hippocampus*, are listed as subjects of different types of protection from over-exploitation and are of particular conservation significance [169]. A set of up to 24 microsatellites has been constructed for cross-species amplification of 15 of species, which will facilitate their further conservation activities. 

## 5. Identification of Management Units and Mixed Stock Analysis

Fishery management aims to determine the maximum catch size, a level that allows exploited populations of aquatic animals to be restored for sustainable harvesting. Marine waters are divided into geographical and administrative areas (management areas) inhabited by theoretically separate stocks. The stocks correspond to spatial management units. The catch limits in Europe (total allowable catches—TACs) are set annually for each management area. There is a growing understanding among fishery managers that the management units should be delineated using not only environmental but also biological criteria, including the genetic structure of stocks and populations. Mismatches between management units and population genetic structure have often been reported [251]. Microsatellites have been used successfully for identification of management units of exploited species, e.g., four species of Pacific salmon [59], freshwater fish, *Piaractus brachypomus*, in the Orinoco and the Amazon basin in South America [116], Korean rockfish, *Sebastes schlegeli* [252], common snook, *Centropomus undecimalis* [253], geoduck clams, *Panopea abrupta* and red sea urchins, *Stronglocentrotus franciscanus*, in British Columbia [254], analysis of relatedness in natural populations of brown trout, *S. trutta*, in Denmark [255,256], parentage analysis and reproductive success at spawning sites of sedentary brown trout, *S. trutta* [257], and impacts of fishery on populations of targeted and by-catch species such as the cod, *G. morhua*, population in the Flamborough Head area, the North Sea [258]. Studies of reproductive success in Atlantic cod revealed that females and males achieved their highest reproductive success when breeding with mates larger than themselves. Therefore, size-selective harvesting may have negative consequences for population recovery due to reductions in the mean body size of commercially exploited marine fishes [259]. 

Overfishing effects on dusky kob, *A. japonicus*, in South Africa [260], stock composition in mixed stock fisheries as in Fraser River (British Columbia, Canada) sockeye salmon, *O. nerka* [261], mixed-stock analysis of Chinook salmon, *O. tshawytscha*, from the Yukon River, Alaska [218], mixed stock analysis of Lake Michigan’s lake whitefish, *Coregonus clupeaformis* [262], mixed-stock analysis of American shad, *Alosa sapidissima*, in two Atlantic coast fisheries, Delaware Bay, USA, and Inner Bay of Fundy, Canada [263], mixed-stock analysis of brown trout, *S. trutta* from the Gulf of Finland [264] have all been studied with microsatellites. Genetic homogenization between the wild Vindelalven salmon population and hatchery stocks of the Angermanalven and Lulealven was observed over 1985–2003, confirmed extensive straying from geographically distant hatchery releases into the wild salmon population and indicated genetic risks associated with large-scale stocking practices in the Baltic Sea [265]. Only rare examples of published reports on selective angling can be found: angling captures more hatchery released and hybrid brown trout in comparison with wild individuals from stocked populations in the Doubs River, Switzerland [266]. 

## 6. Population Genetic Structure over Time

Stability in the genetic structure of populations throughout the age cohorts (juveniles and adults) has been related to a sufficiently large effective population size, which prevents genetic drift over generations, e.g., that detected with microsatellites and mtDNA in wild populations of the Antarctic toothfish, *Dissostichus mawsoni* [267]. Based on microsatellite detection, the following have been reported for some other species: the long-term stability of the genetic diversity of the declined population of the Japanese eel, *Anguilla japonica*, in the north of Taiwan from 1986 to 2007 [268], the temporal stability over the last 45 years of a pike, *Esox lucius* L., stocked population in Stege Nor, Denmark [269], the stability of Atlantic herring, *C. harengus*, in the Baltic Sea and Skagerrak waters over a 24-year period [28], in the Gulf of St. Lawrence in Canada over a 80-year period despite intensive fishing [270], and in wild Australian populations of barramundi, *Lates calcarifer*, over 25 years [271], the genetic composition of cod, *G. morhua*, in the Western Bank over a few years [272], and gilthead sea bream, *Sparus aurata*, in wild samples from the Aegean and Ionian Seas [273]. Historic angling records suggest the occurrence of a drastic decline in the Atlantic salmon population size in the River Eo, Asturia, Spain, during the past two decades; but high levels of diversity found with microsatellites suggest that the population has not been greatly affected by the historical declines and can be expected to recover in the future [274]. 

On the other hand, temporal changes in the genetic structure of wild populations have been reported for brown trout, *Salmo trutta*, before and after population decline and for its stocking with non-local strains of hatchery trout in rivers in Denmark over 60 years [275,276,277,278,279], and for populations of Lake trout, *Salvelinus namaycush*, in the upper Laurentian Great Lakes of North America after their substantial decline in abundance and distribution during the mid-twentieth century and following their recovery with the partial contribution of enhancement from hatcheries [280]. Historical analysis of genetic variation reveals the low effective population size of a northern pike, *Esox lucius*, population and the 8% loss of its heterozygosity over a 32-year period [281], the temporal changes in populations of Atlantic salmon in northern Spain over 20 years [282] and in Denmark for 60 years [283], the introgression of introduced Scottish strains in wild Atlantic salmon populations in southern France assessed through historic scale collections from 1970–1997, and the changes in the genetic composition of in wild rainbow trout populations after the chemical spill in the upper Sacramento River, which generated significant effects over time (1993–1996) on the genetic population structure of rainbow trout throughout the entire upper river basin [284].

## 7. Stocking Effects and Restoration of Wild Populations

Hatchery populations may undergo genome-wide selective sweeps that can affect their fitness and linked neutral loci, such that individuals destined to be released to the wild, should be modified to minimize genetic adaptation to captivity [285]. Temporal genetic variation in the endangered eastern freshwater cod, *Maccullochella ikei*, has been found in the Clarence River system, eastern Australia, using microsatellite DNA markers [286]. Comparison between historical extirpated and restocked populations revealed a significant loss of heterozygosity and allelic richness. Released hatchery-produced material has contributed to the genetic decline in the largest wild *M. ikei* population. This observation demonstrates the adverse effects of stocking programs and the necessity of support from genetic analysis in the design of the management of breeding and stocking strategies, particularly for threatened species [286]. Microsatellite markers revealed very low levels of genetic diversity in the Kootenai River white sturgeon population. The conservation aquaculture program captured 96% of the population’s microsatellite diversity in hatchery-released progeny in only 10 years by using high numbers of broodstock. A panel of 18 microsatellite loci has been validated for parentage analysis [287]. 

Effects of stocking on the genetic integrity of Arctic charr, *Salvelinus*, populations have been found in two lakes in the Bavarian Alpine region [288]. A loss of genetic integrity has been observed in stocked populations of lake trout (*S. namaycush*) from 72 unstocked and stocked lakes in Canada, in which an increase in genetic diversity and a twofold decrease in the extent of genetic differentiation among stocked populations compared to that among unstocked populations has been found [289]. Possible changes in the effective population size of brook charr, *S. fontinalis*, in Québec, Canada, related to long term stocking have been reported [290]. 

Stocked populations were characterized by significant admixture at both population and individual levels, in populations of brown trout, *Salmo trutta* in the Borne River in the Northern French Alps [291], stocked populations on Funen Island, Denmark [292] and populations in tributaries to the Limfjord, Denmark [256]. Admixture was found in populations in which population structure was highly affected by multiple stocking and river diversion in a high mountain national park in Norway [293], in restocked populations in Asturias, Spain [294], and as impact of supportive breeding to enhance populations of salmon in Asturias, Spain [282], sea trout *S. t. m. trutta* in Polish rivers, southern Baltic [295,296], amago salmon, *Oncorhynchus masou ishikawae* in Japan [297], Japanese chum salmon, *O. keta* [298] and in striped bass, *Morone saxatilis* populations in the south eastern USA [299]. 

Enhancement of wild populations has generated changes such as the impact of straying of sea ranched hatchery-reared Atlantic salmon, *S. salar*, on the genetic composition of populations within the small Ellidaar river system in SW Iceland [300], differences in the reproductive success of released natural and hatchery salmon in the Swedish river Dalalven [301,302], differentiation of admixture rates in samples of salmon collected between 1998 and 2006 compared to samples from 1965 to 1987 in France suggesting the similar rising, long-lasting or short-term impacts of stocking with captive-bred fish [303,304,305], and the enhancement of populations of Atlantic salmon in the Connecticut River and Penobscot River, USA [306]. Alleles originating from stocking with non-native, mainly Scottish fishes performed in the 1970s–1990s are present in the contemporary populations of salmon in the River Sella, Spain, which confirms the long term effects of introgression into pristine populations [307]. 

Microsatellite DNA polymorphism evaluation of hatchery-based stock enhancement of black sea bream, *Acanthopagrus schlegelii*, in the South China Sea revealed the significant genetic differentiation of enhanced populations from native populations and the lower genetic diversity of the recaptured released groups of individuals [308]. It has been concluded that the release of cultured juveniles with lowered genetic quality is potentially harmful to the conservation of wild genotypes in native populations. The impact of releases of hatchery-reared fish on natural populations of red sea bream, *Pagrus major*, in Sagami Bay and Tokyo Bay in the Kanagawa Prefecture [309], Kagoshima Bay, Kiusiu [310], Shikoku Island [311], and black sea bream, *A. schlegelii*, in Hiroshima Bay, Japan [312], and the genetic effects of nearly three decades of Murray cod (*Maccullochella peelii peelii*) stocking in five river catchments in southern Australia [313] have been reported. Changes in wild populations have also been caused by releases of Japanese flounder, *Paralichthys olivaceus* [314]. Restocking programs have been developed as a conservation method for a tropical fish, the pacu, *Piaractus mesopotamicus*, because of declines in the number of wild populations in the Tiete and Grande rivers, Brazil, to be accompanied by the genetic monitoring of populations and broodstock to ensure the viability of such programs [315]. 

Microsatellite analysis has contributed to the identification of the sturgeon *Acipenser oxyrinchus* in North America (as opposed to the European *A. sturio*) as a native extinct species in the Baltic Sea and to the genetic control of its imported fry from Canada before the release into the wild in attempts to achieve the restoration of its population in drainages [316,317], restoration of endangered populations of the Adriatic sturgeon (*A. naccarii*) endemic to the North Adriatic region, in the Ticino River Park, Italy [115], and the restocking of endangered populations of dusky grouper, *Epinephelus marginatus*, in the Mediterranean [318], and of white seabream, *Diplodus sargus*, in a fishery reserve in Sicily, Italy [319]. Microsatellites have been used for the estimation of the aquaculture potential of new species such as that of the Korean kelp grouper, *Epinephelus bruneus* [320], yellowtail amberjack, *Seriola lalandi*, in Chile [321] and a flatfish Senegalese sole, *Solea senegalensis* [322]. 

The recovery rate of hatchery-released red drum, *Sciaenops ocellatus*, in some Texas bays and estuaries has been studied [323], as well as that of rock carp, *Procypris rabaudi*— an endemic fish in the upper Yangtze River, China, supplemented with hatchery-produced fish [324]. Assessment of the impact of releasing hatchery-reared juveniles of Pacific abalone, *H. discus* [325], and using molecular pedigree reconstruction to evaluate the long-term survival of out-planted hatchery-reared larval and juvenile northern abalone, *Haliotis kamtschatkana* [326], and assessment of the negative effects of supplementing natural populations of the grooved carpet shell, *Ruditapes decussatus*, on Atlantic coasts of northern Spain using seeds produced in hatcheries [327] have been performed. 

## 8. Escapees’ Impact on Natural Populations

To assess the impact of escapees from cultures and hatcheries, the fitness consequences of the introgression of fast-growing domesticated fish into a wild population were tested [328]. Fry from wild and domesticated rainbow trout (*Oncorhynchus mykiss*) crosses were released into two natural lakes. Parentage analysis was performed using microsatellite loci. The results indicated that domesticated fish can survive as well as wild fish. During the first summer, the fastest-growing crosses had the highest survival, but this trend was reversed after one winter and another summer. The experiment confirms the multigenerational risk of domesticated fish escaping or being released in the case of interbreeding with wild fish. Nile tilapia, *Oreochromis niloticus*, has been introduced throughout Africa, outside its native range for aquaculture purposes, and escapees hybridize with native populations of *Oreochromis* species, which result in negative effects on the conservation of fish biodiversity, aquaculture and capture fisheries in fresh water bodies [329,330]. Microsatellites have been used for identification of escapees of the Oujiang color common carp, *C. carpio* var. *color*, in China [331], analysis of natural hybridization between two species of Andean pupfishes (Cyprinodontidae; *Orestias agassizii* and *O. luteus*) mainly in the Lake Titicaca with implications for local fisheries, stocking and conservation [332], and identification of the occurrence and postulated hatchery origin of hybrids of *Pseudoplatystoma corruscans* and *P. reticulatum* in the Upper Paraná River, in South America [333]. However, modelling gene flow caused by escapees from a few farms simultaneously, revealed that changes detected in a wild population were lower when gene flow was simulated from one farm strain only [334]. 

In the marine environments, net-cage aquaculture poses a risk of the escape of a large number of fish in the case of mechanical damage caused by natural factors (e.g., tsunamis or typhoons) or related to human activities (e.g., shipping and fishing). In Japan, in the mariculture areas, the frequency of gilthead sea bream, *S. aurata*, escapes was estimated to be from 14.1% to 30.2% using microsatellites [335]. Hybridization of escapees in natural populations has been observed. Microsatellites were also used in the identification of escaped farmed gilthead sea bream in the Mediterranean area [273,336,337,338]. Escaped farmed cod, *G. morhua*, were identified in wild populations in Norway [339,340]. Farmed cod from genetically diverse populations grown outside their native range pose the threat of outbreeding depression if they escape and interbreed with wild fish [341]. Microsatellites were used for the assessment of the genetic impact of domesticated farmed escapees on native Atlantic salmon, *S. salar*, populations in Norway [342,343] and Iceland [300], for the assessment of the impact of European Atlantic salmon escapees from hatcheries in Nova Scotia and New Brunswick, Canada on native American, *S. salar*, populations [344], in studies of trophic and epidemiological interactions between salmon farms and the receiving ecosystem including cod preying [345], and for the assessment of the impact of escapees of hatchery European seabass, *Dicentrarchus labrax*, on natural populations in waters around Cyprus [346], and increased relatedness and possible inbreeding in wild populations because of escapees of tropical fish, barramundi *L. calcarifer*, from a sea-cage facility in northern Australia [347]. 

## 9. Comparison of Wild and Hatchery Stocks

Comparisons of feral populations and hatchery stocks using microsatellites have been published for many species (Table 4). 

The lower genetic diversity of cultured populations in comparison with wild populations has been reported for many species, including fish, prawns, sea urchin, oysters, green mussels and abalones [371,372,373,374,375,376,377,378,379,380,381]. 

A small loss of genetic variation in comparison to wild populations was found for Atlantic salmon in a hatchery in Canada [382], and for hatchery brown trout stocks in Finland [383] and in Hungary [384]. Introduced to a Western Australian hatchery, stocks of rainbow trout (*O. mykiss*) are derived from imports from New Zealand, the latter being largely derived from Californian imports in 1883 and also having lower diversity in comparison with wild populations in the north Pacific [385]. Similarly, Tasmanian cultured Atlantic salmon had lower diversity when compared to the progenitor Canadian population [386,387]. A loss of genetic variation was reported in the hatchery stocks of Bleeker’s sheatfish, *Phalacronotus bleekeri* [388], and a loss of genetic diversity (rare alleles) was reported in the cultured stocks of the large yellow croaker, *L. crocea* [389], and starry flounder, *Platichthys stellatus*, in Korea [390], Florida bass, *Micropterus salmoides floridanus* [391], and channel catfish, *I. punctatus*, in farms in Mexico [392], Siberian sturgeon, *Acipenser baeri* [393], *A. gueldenstaedti* and *A. ruthens* in a farm in Poland [394], American paddlefish, *Polyodon spathula*, in Poland [395], barramundi, *L. calcarifer*, hatcheries in Australia [396,397], redclaw crayfish, *Cherax quadricarinatus*, introduced from Australia to culture in China [398], and common carp, *C. carpio*, in the Czech Republic [399]. A loss of genetic variation has also been found in Greek hatchery stocks of the European sea bass, *D. labrax* [400], three tilapia, *Oreochromis*, species cultured in Mexico [401], in hatchery strains of the Pacific abalone, *H. discus hannai* [402], and in cultured Pacific bivalve geoducks (*Panopea generosa*) in Washington state, USA [403]. 

The genetic analysis of pacu broodstocks, *P. mesopotamicus*, used in the stocking program of the Paranapanema River, Brazil, did not confirm reduction in genetic diversity [404]. Sriphairoj et al. [405] concluded from their study that at least 100 brooders (Ne) should be used in practices of managing the critically endangered Mekong giant catfish, *Pangasianodon gigas*, in Thailand. Studies of the banana shrimp, *Fenneropenaeus merguiensis*, in Australia demonstrated the loss of alleles in the mass selection program carried out, even with over 1000 broodstocks being compared with similarly selected but outbred stocks [406]. It is recommended to maintain different and independent lines instead of one line. A high level of genetic variability among Pacific white shrimp, *L. vannamei*, in Pernambuco, Brazil, is sustained by the exchange of breeders between marine shrimp hatcheries [407]. In an attempt to reduce the exploitation of the humpback grouper, *Cromileptes altivelis*, captive breeding has been performed, and the recommendation has been made to increase the effective population size with wild fingerlings in order to avoid diversity reduction detected by microsatellites [408]. Sex-linked microsatellites were found in *Oncorhynchus* [409]. 

Population parameters and the power of 16 microsatellites and 26 SNPs to assign single individuals to their sampling population in wild and farmed stocks of Atlantic salmon (*S. salar*) in Norway were estimated [410]. Microsatellite strain-specific alleles were found. The effectiveness of genetic assignment analysis of populations was almost the same for microsatellites and SNPs (96% of the individuals). The results of analysis of two wild and three cultured Pacific oyster (*C. gigas*) populations with a set of 18 microsatellites (8 genomic simple sequence repeat—SSR; 10 expressed sequences tag (EST)-derived SSR) and 10 EST-derived SNP markers suggest that genomic SSRs and EST-SSRs are more suitable for population genetic analysis than are SNPs [411]. 

Microsatellites are continuously used in studies of genetic diversity in cultured stocks [22,412,413]. 

## 10. Kinship Analysis of Aquacultured Stocks

Lowered genetic diversity in cultured stocks is usually caused by a low effective population size (a reduced number of breeders transmitting their genes to progeny). A loss of genetic diversity decreases the fitness and adaptive potential of the progeny. If the process lasts for some generations, it results in increased relatedness (kinship) among individuals and the effect of inbreeding. Analysis of relatedness has been performed for different strains of rainbow trout, *O. mykiss*, and can be used for the prevention of diversity loss in cultured stocks [414]. Effects of captivity rearing on fitness-correlated traits have been studied in endangered Atlantic salmon, *S. salar*, from the inner Bay of Fundy [415], and on individual reproductive success for fish from the Ste-Marguerite River, QC, Canada [416]. Reproductive success analysis and parentage assignment in culture have been conducted for the optimization of breeding protocols (e.g., by controlled mixing of gamete portions) for white seabass, *Atractoscion nobilis*, and red drum, *Sciaenops ocellatus*, for conservation purposes through stock enhancement programs in California [417]. 

Microsatellite loci have been applied in pedigree tracing of a hatchery strain of Japanese flounder, *P. olivaceus*, to be stocked in natural sea areas [418], parentage analysis and paternity success assessment of turbot, *S. maximus* L. in hatcheries in Spain [419,420], parentage assignment of turbot and rainbow trout in France [421], and Atlantic salmon, *S. salar*, in Ireland [422], parentage analysis and paternity success assessment of cod in Denmark [423], parentage analysis and identification of trait differences in survival and growth among a harvest of communally reared families of Atlantic cod, *G. morhua*, in Canada [424], identification of relatedness and differentiation of hatchery populations of Asian seabass (*L. calcarifer*) broodstock in Thailand [425], parentage analysis of and identification of spawning frequency and timing of brood dams and sires of red drum, *S. ocellatus*, in a marine fish stock-enhancement hatchery in the USA [426], and of European anchovy, *E. encrasicolus*, under a pilot project for aquaculture and enhancement of native populations in Spain [427], analysis of increased genetic relatedness in a hatchery stock in comparison with a wild Senegal sole, *S. senegalensis*, population [428], pedigree classification for giant grouper, *Epinephelus lanceolatus*, broodstock management in Taiwan [429], identification of parental relatedness in a naturalized population of Pacific oysters, *Crassostrea gigas* in Dabob Bay, Washington, USA [430], parentage analysis in Asian seabass for hatchery purposes [431], parentage assessment of blunt snout bream, *Megalobrama amblycephala*, crosses for a freshwater polyculture system in China [432], and greater amberjack, *S. dumerili* [433], estimation of parentage and relatedness in the polyploid white sturgeon, *A. transmontanus* [434], and experimental assessment of genetic tagging with multiplexed microsatellite markers and founder representation in hatchery-reared red drum (*S. ocellatus*) fingerlings used in stock enhancement [435]. A microsatellite-based multiplex PCR panel was constructed that allowed 95% of the offspring to be assigned to a single pair of parents of the meagre *Argyrosomus regius* to support the breeding program in the Mediterranean aquaculture [436].

Microsatellites have been employed in shellfish aquaculture for kinship analysis and genetic variation monitoring in a whiteleg shrimp, *Litopenaeus vannamei*, breeding program [437], identification of parentage markers in the swimming crab, *Portunus trituberculatus* [438], parentage analysis of *H. discus hannai* abalone mixed family farming [439] and in a South African hatchery of *H. midae* abalone [440], genotyping of individual *H. asinina* abalone larvae for parentage assignment in aquaculture in Australia in order to maintain the level of genetic diversity [441], genetic improvement in the clam, *Meretrix meretrix*, by crosses and parentage assignment [442,443], parentage analysis and identification of variation in reproductive success of Pacific oyster, *C. gigas*, in a hatchery in France [444,445], and parentage analysis of different color lineages of scallop, *Patinopecten yessoensis* [446]. 

Genetic analysis of broodstock and progeny of the European sea bass, *Dicentrarchus labrax*, with microsatellies, in aquaculture in the larval stage, when families of progeny had been mixed to start the production cycle, has been conducted to help attain balanced parental contribution [447]. The results from an analysis of spotted seatrout, *Cynoscion nebulosus*, dams and sires in two restoration enhancement facilities in Texas were assessed throughout a spawning year by using parentage analysis based on 12 variable microsatellite loci. That and other studies indicate that reductions in Ne of hatchery- or farm-raised progeny stem primarily from non-contributing dams, suggesting that periodic identification and removal of low-contributing dams from broodfish stocks constitute a critical step toward maximizing the Ne levels of hatchery offspring used in restoration enhancement [448]. The distribution of the F-1-selected breeders into spawning batches should be designed using co-ancestry data, in order to maintain optimal levels of genetic variability in the next generation. This procedure should be repeated for each generation [447]. Based on a simulation study Villanueva et al. [449] reported that, for a set of Atlantic salmon, highly polymorphic microsatellites show, in simulations, that the four most informative loci are sufficient to assign at least 99% of the offspring to the correct parental pair with 100 crosses involving 100 males and 100 females. An additional locus was required for correctly assigning 99% of the offspring when the 100 crosses were produced with 10 males and 10 females. The possibility of selective recovery of founder genetic diversity in aquacultural fish broodstocks has been pointed out [450]. In the case of a limited number of breeders in culture stock, breeding pairs can be matched based on their genetic profiles, obtained with microsatellite loci, in order to assure that only genetically diverse fish are mated [451,452].

Statistical analysis of parentage assignment has been conducted for artificially propagated hatchery fish (removed adipose fin) with parents originating from wild salmon (preset adipose fin) in a population of Chinook salmon, *O. tshawytscha*, from the Wenatchee River, Washington [453]. Simulations demonstrated a lower number of identified parents for hatchery fish in comparison with the number of wild parents. Wild populations of Arctic grayling, *Thymallus arcticus*, from the Lubbock River, Yukon, were sampled for adults and young-of-the-year independently in order to enable the identification of parent–offspring pairs [454]. The genotyping of samples with 38 microsatellites confirmed that a small number of families over-dominated the global number of full-sibs, a phenomenon that is well-known from hatchery stocks. 

A comparison of SNP and microsatellite applicability in parentage and kinship assignment of a wild sockeye salmon (*O. nerka*) population in Alaska demonstrated that the assignment success of 80 SNPs (80 independent alleles) was higher than that of 11 microsatellites (192 independent alleles) but the identification of full-sib groups without parental information from relatedness measures was possible using both marker systems [14]. In a study of hatchery steelhead in the Snake River basin, it was confirmed that a panel of 17 microsatellites was comparable in accuracy in conducting parentage-based tagging (PBT) to a panel of 95 SNPs, and matched that using traditional coded-wire tags (CWT) [455]. The advantages of using microsatellites and SNPs in parentage assignment have been reviewed with an indication that the SNP-based method can benefit from the development of genomics [456]. The parentage analysis with the close-kin mark–recapture (CKMR) method based usually on a large number of SNP loci, has recently been worked out for a few species, such as salmon, thornback ray, *Raja clavate*, and the Pacific white shrimp, *L. vannamei*, with high potential for further applications [457,458,459]. Nevertheless, microsatellites remain the most accessible marker system in the preset day kinship analyses of cultured and wild populations [460,461,462]. 

## 11. Selection of Characters of Choice and Heritability

Selection for a breeding date has been conducted for a coho salmon, *O. kisutch*, population colonizing a new habitat, made accessible by the modification of the Landsburg Diversion Dam, in the Cedar River, Washington, USA [463]. Microsatellites were used for parentage analysis. The offspring of fish arriving earlier to the spawning ground were larger. Larger fish produced more offspring. The hypothesis of ‘pathogen-driven selection in the wild by means of frequency-dependent selection or change in selection through time and space’ has been confirmed by the results of studies of the correlation between the major histocompatibility complex (MHC) class II beta variation and the pathogen infection levels in wild populations of Atlantic salmon, *S. salar*, in Quebec, Canada [464]. Microsatellites were used for monitoring changes in the studied populations. Studies on disease resistance and polymorphisms of major histocompatibility genes, genotypes frequencies and control microsatellite loci in the parr and migrant stages in the wild in Atlantic salmon *S. salar* in Ireland revealed that the additive allelic effects were more likely to determine survival, which highlights the importance of preserving genetic diversity in the wild [465]. White spot disease caused by white spot syndrome virus (WSSV) infection affected shrimp culture throughout the world. Resistant *P. monodon* shrimp identified by microsatellite DNA markers in different seasons were collected from natural populations along the entire east coast of India with the aim to obtain broodstock to prevent a repeated outbreak of white spot disease in a hatchery [466,467]. 

Selection programs have been executed with the aim to improve the quality of cultured fish and shellfish for releases to the wild or for consumption. Microsatellites were employed in selection in the larval stage for the faster growth of adults of Asian sea bass (barramundi), *L. calcarifer* [468], selection for growth in the European sea bass, *D. labrax* [469], genetic comparison of different hatchery strains of rainbow trout, *O. mykiss*, from the Northwest in the USA selected for growth and immunological response [470], commercial selection using DNA parentage assignment in rainbow trout aquaculture [471], confirmation of the ‘hypothesis of a dominant mutation mechanism’ as a possible cause of rib and vertebral deformities found in farmed rainbow trout [472], and a study of the impact of domestication on the stress response and immune modulation in Eurasian perch, *P. fluviatilis* [473]. Selection for performance in salt water and *S. salar* salmon broodstock development for hatcheries in New Brunswick, Newfoundland and Labrador in Canada, included genetic analysis, an estimation of heritability of bacterial kidney disease, sea lice, growth, fillet yield and deformities [474]. Immune-related loci were identified as *F_ST_* outliers in pairwise comparisons of samples at a 10-fold higher frequency than that of neutral loci in Atlantic salmon, which means that well characterized immune-related loci as well as neutral loci (microsatellites) in cultured species can be useful when disease control and prevention is a goal [475]. Assessment of the genotype by environment interaction for the growth of sole (*S. solea*) in an intensive recirculation aquaculture system (RAS) and in a semi-natural outdoor pond (POND) has shown low genetic correlations for growth between environments, which implies that the best genotypes in an intensive aquaculture environment are not necessarily the best genotypes in more natural environments such as ponds [476]. 

Microsatellites were used for parentage identification in studies of heritability and genotype by diet interactions of European sea bass (*D. labrax*) affecting fish weight and size at age [477] and flesh quality, studies of the heritability of cold tolerance under progressive temperature decrease, and of acute cold-stress tolerance in red drum, *S. ocellatus*, in southern USA [478,479]. Arctic charr, *S. alpinus*, eggs were obtained for research from two North American sources, an eastern (Fraser River, Canada) and western (Bristol Bay, Alaska, USA) stock and fishes were mixed and grown for 2 years in tanks [480]. Genetic correlations between body size traits were highly positive and significant. The genetic correlation of fillet fat with fillet color was positive and significant. The eastern stock was composed of an admixture of two sources; the commercial stock was composed of three different sources, and the western stock was composed of three to four source populations as inferred from 480 microsatellites. The heritability of Atlantic salmon flesh color traits was low to medium with carotenoid content in the flesh exhibiting the lowest additive genetic variation [481]. The heritability of morphological abnormalities in gilthead seabream, *Spares aurata*, has been shown to be significant [482]. Microsatellite-based parentage pedigree analysis is continuously used in the present day research of the heritability of different traits in fish and shellfish species [483,484,485]. 

## 12. Gynogenesis Assessment

Gynogenesis involves fertilization of eggs with UV-irradiated sperm (with DNA deactivated) exposed to the cold or chemical shock followed by pressure treatment [486,487]. Inhibition of the mitotic cleavage results in homozygous double haploid (mitotic) gynogenetic progeny. Maturing female eggs fertilized with irradiated sperm and interrupted meiosis (meiotic gynogenesis) are clones of the same female sex. Gynogenesis occurs very rarely in wild populations, but is used for research and culture purposes to obtain all-female specimens. Such monosex females are more economical in commercial culture because of their faster growth. Microsatellites have often been used for genogenetic assessment, e.g., in the case of African catfish, *Clarias gariepinus* [488], in the case of gynogenetic diploids being generated in order to map centromeres of walking catfish, *Clarias microcephalus* [489], large yellow croaker, *Pseudosciaena crocea* [490] (Miao et al., 2015), turbot, *S. maximus*, in Spain [491], half-smooth tongue sole, *Cynoglossus semilaevis*, [492], shortnose sturgeon, *Acipenser brevirostrum* Lesuere [493], Siberian sturgeon, *A. baeri* Brandt [494,495], and starlet, *A. ruthens* [496], in studies of the sex determination system in ship sturgeon, *Acipenser nudiventris*, using meiotic gynogenesis [497], in the assessment of gynogenesis in stellate sturgeon, *Acipenser stellatus* [498], wels catfish, *Silurus glanis* [499], red crucian carp [500], and Japanese flounder, *P. olivaceus* [501], and in the analysis of gynogenetic diploids induced by heterologous sperm in *Chlamys farreri* [502]. 

Microsatellites were used for assessment of androgenesis (originating from males - all-males), e.g., in the loach, *Misgurnus anguillicaudatus* [503], amago salmon, *O. masou ishikawae* [504], and in large yellow croaker, *P. crocea* [505]. Microsatellites were useful in the analysis of ploidization, including evolutionary polyploidy, which occurred through the hybridization of common carp, *C. carpio*, approximately 12 MYA [506], functional hexaploidy in the shortnose sturgeon, *A. brevirostrum*, ‘functional genome reduction’ in other species of sturgeons [507], natural ploidization and induced gynogenesis in the loach, *M. anguillicaudatus*, and blunt snout bream, *M. amblycephala* [508,509], parental assignment of natural triploids, diploids and laboratory induced gynogensis of loaches, *M. anguillicaudatus*, in the Hokkaido island, Japan [510], analysis for laboratory and aquaculture purposes of ploidy levels in *Acipenser* hybrid larvae [511] and tests of Mendelian segregation in bester—a hybrid of beluga, *Huso huso* L., and sterlet, *Acipenser ruthenus* L., in the fourth generation [512]. 

Microsatellites have been found to have applications in research concerning the induction of triploids in cod, *G. morhua* [513], induction of triploids and gynogenesis in Senegalese sole, *S. senegalensis*, for aquaculture [322], pressure and cold shock induction of meiotic gynogenesis and triploidy in the European sea bass, *D. labrax* [514], parental assignment in triploids of Pacific oysters, *C. gigas*, for improved selection for fast growth in aquaculture [515], production of tetraploids [73], characteristics of spotted mandarin fish, *Siniperca scherzeri*, and X (female) mandarin fish, *S. chuatsi* (sic), F1 and F2 hybrids [516] (Li et al., 2014), assessment of hybrids of *Haliotis rufescens* with *H. discus hannai* produced in Chile [517] (Lafarga de la Cruz et al., 2010) and *H. discus hannai* Ino with *Haliotis gigantea* in China [518] (Luo et al., 2010), and the hybridization of the swimming crab, *P. trituberculatus*, distributed in the coastal waters of Asia-Pacific with the aim to increase its performance in hatchery [519]. Sex-linked microsatellite alleles were found in gynogenetic individuals of turbot, *S. maximus* [520]. Microsatellites are applied in assessment in the most recent research concerning gynogenesis, androgenesis and ploidization [521,522,523]. 

## 13. QTL Identification

Tolerance to environmental stress factors such as temperature, salinity, low oxygen (hypoxia), changes in pH, food availability and biological factors, resistance to viral and bacterial pathogens and parasites determine survival under natural conditions and productivity under aquaculture aconditions. Genomic regions associated with the trait of interest can be mapped in order to identify quantitative trait loci (QTL). Microsatellites have been commonly used successfully for finding the linkage of molecular markers with QTL. Subsequently, such markers can be used in marker-assisted selection (MAS) in aquaculture. Analysis of genomic sequences and genetic differentiation of associated tandem repeat markers (microsatellites) in the growth hormone somatolactin and insulin-like growth factor-1 genes of the sea bass, *D. labrax*, proved that gene-associated markers are more efficient than formerly used anonymous microsatellite loci at providing a clear picture of genetic differentiation [524]. Genetic linkage analysis is an effective method for identifying quantitative trait loci (QTL) associated with resistance to a disease [525]. Microsatellies have been intensively applied in the construction of genetic linkage maps, e.g., for salmon, *S. salar* [526,527], for Arctic char (*S. alpinus*) using two backcrosses between genetically divergent strains [528], a common carp, *C. carpio* [529,530], European sea bass, *D. labrax* [531], gilthead sea bream, *S. aurata* [532], and Pacific abalone *H. discus hannai* [533]. 

A genetic map was developed for a population of European sea bass with the use of ove 90 microsatellites and enabled the finding of two QTL for body weight, six QTL for morphometric traits and three suggestive QTL for stress response [534]. Genetic linkage maps have been constructed for red drum, *S. ocellatus* [535,536], barfin flounder, *Verasper moseri*, spotted halibut, *Verasper variegatus* [537], Atlantic halibut broodstock management in Canada including tentative QTL for pigmentation, body size and eye migration [538], and for turbot, *S. maximus*, and one possible QTL associated with body length was detected [539]; however, the observation of a high mean variation between traits among families made it difficult to evaluate QTL effects [540]. Microsatellite genetic linkage maps were elaborated for brill, *S. rhombus*, for a preliminary study on growth-related QTL for body weight, length and Fulton’s condition factor [541] and for four tilapia species [542]. Located on the maps were QTL found to be related to sex determination in Mozambique tilapia [543], QTL for the spawning date of Coho salmon, *O. kisutch* [544], QTL influencing early maturation [545] and upper thermal tolerance in outbred strains of rainbow trout, *O. mykiss* [546]. Thirteen QTL markers for spawning time representing seven linkage groups were found, and eight markers from five linkage groups showed consistent effects in two sampling years, which suggests this trait is highly polygenic [547]. for a high heritability of body mass and the condition factor and a moderate heritability of the age of sexual maturity of males was found in two cultured strains (Rainbow Springs and Spring Valley) of rainbow trout [548]. Faster growing individuals were more likely to mature at two years of age than slower growing individuals. The location of microsatellite markers of body mass QTL in linkage groups was reconfirmed and new ones were detected. Seven tentative and three significant QTL were detected in families that exhibited high or low plasma cortisol concentrations in response to crowding stress in rainbow trout culture production.

QTL for morphometric traits in gilthead seabream, *S. aurata* [549,550,551], and QTL for short-duration vigorous swimming movements in common carp (*C. carpio*) based on LDH Activity [552] have been found. A first-generation microsatellite-based linkage map was created for the Chinese mitten crab, *Eriocheir sinensis*, and used for QTL detection, and nine growth-related QTL for body length, width and weight were mapped to seven linkage groups [553]. Additionally, two QTL were identified to be associated with sexual precocity. The linkage maps and the identified QTL will be valuable for marker-assisted selection breeding programs. Genetic linkage maps were elaborated for the Pacific lion-paw scallop, *Nodipecten subnodosus*, and putative QTL were identified for morphometric traits [554], QTL for pearl quality traits in the freshwater triangle pearl mussel, *Hyriopsis cumingii* in China [555], and for the growth rate in the blacklip abalone, *H. rubra* from Australia [556] were found, QTL for size in Bay scallop, *Argopecten irradians* [557], was found, and a linkage map for Chinese shrimp, *Penaeus (Fenneropaeneus) chinensis* [558] was constructed. A total of 159 microsatellite markers were selected from genetic linkage maps of Japanese flounder, *P. olivaceus*, and F-1 progeny of crosses between disease-resistant and disease-susceptible parents were used for the detection of QTL associated with resistance to Streptococcal disease (streptococcosis) caused by *Streptococcus iniae* [525]. These candidate QTL regions have been found. QTL with significant effects on infectious pancreatic necrosis (IPN) resistance in Atlantic salmon, *S. salar*, using a genome scan [559], and in rainbow trout, *O. mykiss* [560] (Ozaki et al., 2001), were found, and candidate QTL for infectious salmon anemia (ISA) resistance in Atlantic salmon, *S. salar* [561], were found. Bacterial cold water disease (BCWD) causes significant economic loss in salmonid aquaculture, and previously, genetic variation was detected in survivors following challenge with *Flavobacterium psychrophilum*, the causative agent of BCWD in rainbow trout, *O. mykiss* [562]. The nine major QTL identified in that study are candidates for fine mapping to identify new markers that are tightly linked to disease resistance loci for use in marker-assisted selection strategies. 

Backcrosses of rainbow trout and steelhead (*O. mykiss*) were used to construct a linkage map and to identify associations between molecular markers, QTL determining resistance to infectious hematopoietic necrosis virus (IHNV) and associations were observed for four of the markers [563]. Candidate QTL were found for resistance to viral nervous necrosis disease (VNN) in Asian seabass [564], tentative QTL were found for resistance of gilthead sea bream to fish pasteurellosis caused by a bacterial pathogen, *Photobacterium damselae*, subsp. *Piscicida* [565], microsatellite loci were associated with growth-related traits in the Manila clam *R. philippinarum* [566], QTL connected with stage-specific inbreeding depression were found in the Pacific oyster, *C. gigas* [567], and QTL were found in the flat oyster, *O. edulis*, for resistance to a parasite, *Bonamia ostreae* [568]. The discovery of QTL for resistance to summer mortality and OsHV-1 infection in the Pacific oyster (*C. gigas*) opens new possibilities of selection for resistance to oyster herpesvirus, OsHV-1 [569], and applications in the epidemiology of livestock species, such as in flat oysters, *O. edulis*, in France and around the world, with worldwide mortality being observed since 2008 [570,571,572]. A characterization of novel EST-SSR markers and their correlations with growth and nacreous secretion traits has been performed for the *Pinctada martensii* pearl oyster, the primary cultured species of marine pearls in southern China [573]. Twenty-nine novel polymorphic microsatellite markers were developed to facilitate marker-assisted selection in the genetic improvement of this species. 

Several QTL related to phenotypic variation have been identified in the salmon, *S. salar*, genome. Population differentiation and assessment of linkage disequilibrium in chromosomes containing QTL for body weight, infectious pancreatic necrosis resistance and infectious salmon anaemia resistance to detect the selection history at the genomic level in Atlantic salmon have been used by Martinez et al. [574]. They demonstrated that the body weight QTL (marker SSA0343BSFU on chromosome 3) has been under directional selection. This marker is physically mapped near the coding sequence of DVL2 (for segment polarity protein disheveled homolog DVL-2) and is a good candidate gene related to the quick response to selection for growth. However, only low diversifying selection was found in the QTL associated with infectious pancreatic necrosis and infectious salmon anemia resistance. Due to their rather high selection intensity, individual loci may undergo indirect selection and increased inbreeding. Therefore, it can be concluded that artificial selection has inflicted significant changes to the Atlantic salmon genome, validating the QTL in cultured salmon populations used in industry production resulting from the recent selection history [574]. This conclusion can be ubiquitously applied to other cultured aquatic species. 

Microsatellites have been used also in studies of phenotypic changes in transgenetic *C. carpiovar var.* Jian [575]. Microsatellites can also be useful markers for the identification of novel transposable elements, such as the major histocompatibility complex (MHC) class I region of a teleost, medaka, *Oryzias latipes* [576]. Population genetic differentiation has been found in parasitic sea lice (*Lepeophtheirus salmonis*) on Atlantic and Pacific salmonids from wild and farmed fish, by analyses of microsatellite DNA [577]. Microsatellite identification coupled with SNP discovery have been used recently for mapping QTL for such traits as cold-tolerance and disease resistance of farmed tilapia [578,579], and for identifying the occurrence of skeletal abnormalities in gilthead seabream [580]. Nevertheless, it should be noted that SNP markers are increasingly used for research related to QTL. 

## 14. Conclusions

Simple sequence repeats (SSR—microsatellites) have been found to have a variety of applications in genetic research related to natural populations and cultured stocks over the past 30 years. Microsatellites have been known as hypervariable neutral molecular markers with the highest resolution power in comparison with any other markers. The largest number of publications concerned the detection of the genetic structure of pristine populations in natural conditions, identification of invasive species, detection of evolutionary management units for conservation genetics, identification of fishery management units and mixed stock analysis, effects of stocking or escapes of farmed fish and their interactions with natural populations, analysis of hatchery stocks including kinship and parentage analysis, assessment of gynogenesis, selection for characteristics of choice and heritability in the wild and identification of quantitative trait loci (QTL). However, the discovery of a new type of molecular marker—single nucleotide polymorphism (SNP) has put the existing applications of microsatellites to the test. Both types of markers have become more available with the development of genomics. In population related studies, several (14–20) microsatellite loci correspond to about 200 independent alleles, which can ensure good accuracy when distinguishing populations. Recently, these numbers tend to be increased by the application of genomic sequencing of expressed sequence tags (ESTs), and using only the most informative loci for genotyping, depending on the aims of research. However, the number of SNP loci can be increased to thousands, which makes it possible to obtain more detailed and precise information on populations. Because SNPs are neutral or outliers (possibly under natural selection), they are able to uncover more effectively both differentiation among populations and the various processes taking place in them, such as introgression, hybridization or the reconstruction of demographic changes. It is noticeable that the number of recent papers related to population level processes associated with microsatellites has decreased, whereas of those using SNPs has increased. Nevertheless, microsatellites can be considered to be superior markers in such topics as kinship and parentage analysis in cultured and natural populations, assessment of gynogenesis, androgenesis and ploidization. For other purposes, microsatellites coupled with SNPs for mapping QTL will remain feasible. Microsatellites will continue to be used in studies of genetic diversity in cultured stocks, and also in the research of natural populations as an economically advantageous research technique.

## Figures and Tables

**Table 1:** Examples of applications of protein allozymes in studies of aquatic species.

Topic	Species	Region	References
**Genetic structure of wild populations**			
	European anchovy, *Engraulis encrasicolus*	Adriatic, NE Atlantic	[23]
	sea trout, *Salmo trutta*	Norway, NE Atlantic	[24]
		Baltic Sea, NE Atlantic	[25]
	Atlantic salmon, *Salmo salar*	British Isles, NE Atlantic	[26]
		NE Atlantic	[27]
	Atlantic herring, *Clupea harengus*	Sweden, NE Atlantic	[28]
	red mullet, *Mullus barbatus* and *M. surmuletus*	Mediterranean Sea, NE Atlantic	[29]
	mullets, *M. cephalus*, *M. soiuy*, *Liza ramada*, *L. aurata*, *L. abu*, *L. saliens*, *L. carinata*, *Chelon labrosus*, *C. labrosus*	Mediterranean Sea, NE Atlantic	[30,31,32]
	tuna, *Auxis thazard*, *A. rochei*, *Euthynnus affinis*, *Katsuwonus pelamis*, *Sarda orientalis*, *Thunnus tonggol*, *T. albacares*	Indian Ocean	[33]
	chum salmon, *Oncorhynchus keta*	Alaska, N. Pacific	[34]
	chum salmon, *Oncorhynchus keta*	Islands of Japan, NE Pacific	[35]
	atlantic salmon, *S. salar*, brown trout, *S. trutta*, and their hybrids		[36]
	sockeye salmon, *Oncorhynchus nerka*	Islands of Japan, NE Pacific	[37]
	Brook charr, *Salvelinus fontinalis*	Newfoundland, NW Atlantic	[38]
	Atlantic cod, *Gadus morhua*	Norway, NE Atlantic	[39]
	brown trout, *S. trutta*	Europe, NE Atlantic	[40]
	horse mackerel, *Trachurus trachurus*	Mediterranean, NE Atlantic	[41]
	Atka mackerel, *Pleurogrammus monopterygius*	Aleuts, N Pacific	[42]
	turbot, *Psetta maxima*, brill, *Scophthalmus rhombus*	Europe, NE Atlantic	[43]
	red king crab, *Paralithodes camtschaticus*	Bering Sea, Gulf of Alaska, N. Pacific	[44]
	Norway lobster, *Nephrops norvegicus*	North Sea, Aegean Sea, NE Atlantic	[45]
	clam, *Macoma balthica*	Pacific and Atlantic coast, N America	[46]
	coot clam, *Mulinia lateralis*	NW Atlantic	[47]
	blue mussel, *Mytilus edulis*	NW Atlantic	[48]
	mussels *M. edulis*, *M. trossulus*, *M. galloprovincialis*	Northern and Southern Hemispreres	[49]
**Phylogenetic relationships**			
	*Salvelinus alpinus*, *S. malma*, *S. confluentus*, *S. leucomaenis*	North America	[50]
	23 cyprinid species (Alburninae and Leuciscinae)	Central Europe	[51]
	hake *Merluccius australis*, *M. hubbs*	SW Atlantic	[52]
	*Sepia officinalis*, *S. orbignyana*, *S. elegans*	Iberian Penisula, NE Atlantic	[53]
**Conservation genetics**			
	brown trout, *S. trutta*	Iberian Penisula, NE Atlantic	[54]
	sockeye salmon, *O. nerka*	Kenai River drainage, Alaska, N Pacific	[55]
	salmonids	E Pacific	[56]
**Changes in populations associated with fishery exploitation, management units, stocking**			
management units	salmon, *S. salar*	N Baltic, NE Atlantic	[57]
mixed stock analysis	chum salmon, *O. keta*	Yukon River, Alaska, NE Pacific	[58]
fisheries management	chinook salmon, pink salmon, chum salmon	NE Pacific	[59]
stocking and escapees from aquaculture	native cutthroat trout, *Oncorhynchus clarki* introgressed with stocked rainbow trout, *O. mykiss*	Great Basin, Oregon, NE Pacific	[60]
effects of stocking	brown trout, *S. trutta*, *morpha fario*	Mediterranean	[61]
management units—stocking	black bream, *Acanthopagrus butcheri*	S Australia	[62]
interbreeding between the farmed escapes strain and the wild population	*S. salar*	Glenarm River, N Ireland, NE Atlantic	[63]
impact of the accidental and deliberate introduction of non-native salmonids on the genetic make-up of natural populations	*S. salar*, *S. trutta*	Europe, N Atlantic	[64]
**Aquaculture genetics**			
gynogenesis	prucian carps, *C. gibelio*	Ukraine, Europe	[65]
less genetic diversity in hatchery stocks in comparison with natural populations	turbot, *Scophthalmus maximus*	Iberian Penisula, NE Atlantic	[66]
hatchery rearing and implications of the use of reared fish in enhancement programmes	salmon, *S. salar*	N Ireland, NE Atlantic	[67]
selected lines for growth had lower mean heterozygosity, lower percentages of polymorphic loci, and fewer alleles per locus than control lines; alleles correlated with growth rate	channel catfish, *Ictalurus punctatus*	Alabama, USA, N. America	[68]
spontaneous gynogens, aneuploids, or possibly incompatible with regulatory loci, hybridization, and triploids	Coho, *Oncorhynchus kisutch*, chum, *O. keta*, chinook, *O. tshawytscha*,	Wshington, E Pacific	[69]
loss of genetic variability in hatchery stock over 16 years	brown trout, *S. trutta*	Finland, Baltic, NE Atlantic	[70]
reduction in polymorphism in hatchery stock introduced to Chile	Coho salmon, *O. kisutch*	Chile, SE Pacific	[71]
Genetic improvement	Pacific oyster, *Crassostrea gigas*	Australia imported to hatcheries from Japan Tasmania, Australia	[72,73]

**Table 2:** Examples of mitochondrial DNA applications in studies of aquatic species.

Topic	Species	Region	References
**Phylogenetic and phylogeographic analyses**			
phylogeography	oysters *C. gigas*, *C. angulata*, *C. sikamea*, *C. ariakensis*, *C. hongkongensis*	China, Asia	[89]
phylogeography and glacial refugia	*M. edulis*, *M. trossulus*, *M. galloprovincialis*	Europe	[90]
phylogeography and glacial refugia	schizothoracine fishes	Tibet, Asia	[91]
phylogeography	yellowtail amberjack, *Seriola lalandi*	S Atlantic, Pacific	[92]
phylogeography	brown trout, *S. trutta*	Europe	[93,94]
phylogeography	bivalve pustulose ark, *Anadara tuberculosa*	N South America, Pacific	[95]
phylogeography	black scraper, *Thamnaconus modestus*	East China and Japan Sea	[96]
phylogeography	hilsa shad, *Tenualosa ilisha*	N Indian Ocean	[97]
**Population differentiation studies**			
breakpoint between Atlantic and Mediterranean populations	greater amberjack, *Seriola dumerili*	Mediterranean, NE Atlantic	[98]
large scale differentiation	chum salmon, *O. keta*	Pacific Rim, N Pacific	[99,100]
postglacial colonization	brown trout, *S. trutta*	Central Europe, NE Atlantic	[101,102]
MtDNA of *M. edulis* in *M. trossulus*	mussel, *Mytilus trossulus*	Baltic, Europe	[103,104]
	European anchovies, *E. encrasicolus*	Mediterranean Sea, NE Atlantic	[105]
	mussel, *Mytilus galloprovincialis*	Central East Mediterranean	[13]
no population differentiation	American eel, *Anguilla rostrate*, European eel, *Anguilla anguilla*	N Atlantic	[106]
differences between northern and southern populations	horseshoe crab, *Limulus polyphemus*	NW Atlantic	[107]
differentiation between Alaska and Canada	*Oncorhynchus tshawytscha*	NE Pacific	[108]
	cod, *G. morhua*	E Atlantic	[109]
cncordance alloz, mtDNA and nuclear DNA	sockeye salmon, *O. nerka*	Alaska, N Pacific	[74]
	bay scallop, *Argopecten irradians*	NW Atlantic	[110,111]
MtDNA of herring	Atlantic herring *C. harengus*, Pacific herring *C. pallasii*	N Europe, NE Atlantic	[112]
	Chinese mitten crab, *Eriocheir sinensis*	China, Asia	[113]
**Conservation genetics**			
operational taxonomic units for conservation of endemic species	white-clawed crayfish, *Austropotamobius italicus*	N Italy, Europe	[114]
endemic; restitution of population by stocking	Adriatic sturgeon, *Acipenser naccarii*	Adriatic, Mediterranean Sea	[115]
delimitation of evolutionarily significant units	fish, *Piaractus brachypomus*	Amazon and Orinoco basins, S America	[116]
endemic; stock enhancement	fish, honmoroko *Gnathopogon caerulescens*	Japan	[117]
endemic; stock enhancement	cyprinid fish, *Platypharodon extremus*	Tibet, Asia	[118]
management units	huchen, *Hucho hucho*	Slovenia, Europe	[119]
	wedge clam, *Donax trunculus*	Iberian Peninsula, NE Atlantic	[120]
**Species identification and taxonomy**			
	rougheye rockfish, *Sebastes aleutianus*	Alaska, N Pacific	[78]
species and hybrids	European flounder, *Platichthys flesus*, plaice, *Pleuronectes platessa*	Baltic, NE Atlantic	[121]
species and hybrids	*Oreochromis niloticus*, *Tilapia zillii*, *O. aureus*, *Sarotherodon galilaeus*	rivers in S China, Asia	[122]
	thinlip grey mullet, *L. ramada*	Oder river, Poland, Europe	[123]
**Introgression from restocking**			
	brown trout, *S. trutta*	Iberian Peninsula, Mediterranean, Europe	[124]
introgression depends on environmental conditions in streams	brown trout, *S. trutta*	central Italy, Europe	[125]
low introgression	brown trout, *S. trutta*	SE Europe	[94]
changes in mtDNA haplotype frequency	red sea bream, *Pagrus major*	Kagoshima Bay, Japan, Asia	[126]
low impact	Japanese Spanish mackerel, *Scomberomorus niphonius*	Seto Inland Sea, Japan, Asia	[127]
**Introductions and invasions**			
two main clusters suggest secondary introduction inside Europe	Pacific oyster, *C. gigas*	Europe	[128]
origin: France	pike-perch, *Sander lucioperca*	Tunisia, N Africa	[129]
identification of invasive species of tilapia originating from aquaculture	tilapia	China, Asia	[122]
identification of invasive species of tilapia originating from aquaculture	tilapia	Japan, Asia	[130]
**Studies related to fisheries**			
fisheries management and conservation	12 species	Baltic, NE Atlantic	[131]
low usefulness	Pacific salmons	NW Pacific	[59]
potential application in mixed stock analysis	chum salmon, *Oncorhynchus keta*	Yukon river, NW Pacific	[58]
taxa identification in the commercial marine fishery	Chondrichthyes (sharks, rays)	Indian Economic Zone, N Indian Ocean	[132]
elasmobranch species identification	36 elasmobranch species	Malta, Mediterranean, Europe	[133]
**Aquaculture**			
polymorphism in breeding stocks	rainbow trout, *Oncorhynchus mykiss*	Finland, Europe	[134]
Hybridization experiments	abalones *Haliotis discus discus*, *H. madaka*, *H. gigantea*.	Japan, Asia	[135]
lower polymorphism in cultured stocks	giant freshwater prawn, *Macrobrachium rosenbergii*	China	[136]
assessment of genetic diversity in aquaculture strains	common carp, *Cyprinus carpio*	cental Europe	[17,137]
analysis of interspecific hybrid developmental stages	croakers, *Larimichthys crocea* x *L. polyactis*	China, Asia	[138]
**Seafood testing and forensics**			
identification of shark species in food products	hammerhead shark, *Sphyrna lewini*, basking shark, *Cetorhynus maximus*		[139]
fish product mislabeling (COI)	fish species	France, Europe	[140]
barcoding and fish mislabeling	fish species	N America	[141]
mislabeling of food products	seafood	Europe	[142]
fish product mislabeling	fish species	Germany, Europe	[143]
mislabeling of products	common sole, *Solea solea*	Germany, Europe	[144]
mislabeling of products	fish species	Tasmania, Australia	[145]
mislabeling	sturgeon caviar	Austria	[146]
mislabeling of sushi components	tuna, *Thunnus* sp., eel, *Anguilla* sp.	England, Europe	[147]
tracing illegal aquatic wildlife trade	fish and mammals	Philippines	[148]

**Table 3:** Examples of species with population genetic structures found using microsatellites.

Species	Region	References
rainbow trout, *Oncorhynchus mykiss*; pink salmon, *O. gorbuscha*; chum salmon, *O. keta*; coho salmon, *O. kisutch*; sockeye salmon, *O. nerka*; chinook salmon, *O. tshawytscha*; bull trout, *Salvelinus confluentus*	Elwha River, Waschington, USA, North America	[178]
brook charr, *S. fontinalis*	La Mauricie lakes, Canada, N America	[179]
brown trout, *S. trutta*	North Atlantic, Mediterranean, Europe	[180]
Atlantic salmon, *S. salar*	Iceland, Norway and Ireland, Europe Nova Scotia, Canada, Noth America	[181,182,183]
broad whitefish, *Coregonus nasus*	Mackenzie River, Canada, North America	[184]
Arctic charr, *S. alpinus*	Labrador, Canada, Nunavut, Alaska, North America	[185]
mulloway, *Argyrosomus japonicus*	Southern Australia	[186]
Curimbatá, *Prochilodus lineatus*	Paraná River basin, South America	[187]
*Salminus franciscanus*, *Brycon orthotaenia*	São Francisco River system, Brazil, South America	[188]
red snapper, *Lutjanus campechanus*	Gulf of Mexico, USA, North America	[189]
European hake, *Merluccius merluccius*	North Atlantic, Mediterranean, Europe	[190]
cod, *G. morhua*	North Atlantic, Europe	[39,191,192,193,194]
Pacific herring, *C. pallasi*	Bering Sea and Alaskan waters, N Pacific	[195]
Atlantic mackerel, *Scomber scombrus*	North Atlantic	[196]
yellowfin tuna, *Thunnus albacares*	Central West Pacific	[197]
native cobia, *Rachycentron canadum*	Gulf of Thailand and Andaman Sea, Southern Asia	[198]
Atlantic sturgeon, *Acipenser o. oxyrinchus*	Atlantic coast of North America	[199]
Persian sturgeon, *Acipenser persicus*	Caspian Sea, South Eastern Asia	[200]
black carp, *Mylopharyngodon piceus*	Yangtze River, China, Asia	[201]
European perch, *Perca fluviatilis*	Wulungu and Jili lakes, Kalaeerqisi River, North-West China, Asia	[176]
Nile tilapia, *O. niloticus*	Kenya, Africa	[202]
fish hardyhead, *Craterocephalus fluviatilis*	Murray–Darling Basin, south-eastern Australia	[203]
catfish, *Pseudoplatystoma magdaleniatum*	Magdalena and Cauca rivers, Colombia, South America	[204]
catfishes, *Pseudoplatystoma corruscans*, *P. reticulatum*	Paraguay, Parana, and Uruguay River basins, South America	[205]
*Hypophthalmus donascimientoi*	Solimões, Amazon River, Brazil, South America	[206]
greater amberjack, *Seriola dumerili*	Atlantic and Mediterranean	[98]
white oci, *Seriola rivoliana*	Mediterranean	[98]
longnose skates, *Zearaja chilensis*, *Dipturus trachyderma*	Chile, South America	[207]
longfin squid, *Loligo pealeii*	North West Atlantic	[208]
sea cucumber, *Holothuria mammata*	Mediterranean and Atlantic	[209]
Abalone, *Haliotis asinina*	Heron Reef, Queensland, Australia	[210]
flat oyster, *Ostrea edulis*	Europe	[211]
blue mussel, *Mytilus chilensis*	Southern Chile, South America	[212]

**Table 4:** Comparison of wild populations and hatchery stocks based on the use microsatellites.

Species		Region	References
black scraper	*Thamnaconus modestus*	Korea, Asia	[348]
mi-iuy croaker	*Miichthys miiuy*	Korea, Asia	[349]
spotted sea bass	*Lateolabrax maculatus*	Korea, Asia	[350]
sea bass	*D. labrax*	Mediterranean, Europe	[351,352]
gilthead seabream	*Sparus aurata*	Greece, Europe	[353,354]
turbot	*Scophthalmus maximus*	Ireland and Norway, Europe	[355]
olive flounder	*Paralichthys olivaceus*	Korea, Asia	[356]
olive flounder	*P. olivaceus*	Japan, Asia	[357]
brown trout	*Salmo trutta*	Czechs, Slovakia, Europe	[358]
sea trout	*S. trutta*	Poland, Europe	[359]
sea trout	*S. trutta*	River Dalalven, Sweden, Europe	[360]
Atlantic salmon	*S. salar*	North Europe, Canada, North America	[361,362,363,364]
steelhead	*O. mykiss*	Washington, Pacific, USA, North America	[365]
Arctic charr	*Salvelinus alpinus*	North America	[185]
striped catfish	*Pangasianodon hypophthalmus*	Vietnam, Thailand, Asia	[366,367]
tilapia	*Oreochromis*	South-West Pacific, Fiji, Asia	[368]
Nile tilapia	*O. niloticus*	Ghana, Africa	[369]
Atlantic sturgeon	*A. oxyrinchus*, *A. sturio*	USA, Central Europe	[370]
	*Colossoma macropomum*	Brazil, South America	[371]
prawn	*Penaeus monodon*	Malaysia, Asia	[372,373]
marine shrimp	*Litopenaeus vannamei*	Brazil, South America	[374]
giant freshwater prawn	*Macrobrachium rosenbergii*	USA, Hawaii, Israel, India, Myanmar	[375]
Japanese sea urchin	*Strongylocentrotus intermedius*	China, Asia	[376]
oyster	*Crassostrea virginica*	Chesapeake Bay, USA, Northe America	[377]
Pacific oyster	*C. gigas*	Australia, France, Korea, Japan, Asia	[378]
green mussel	*Perna viridis*	Southeast Asia	[379]
abalone	*Haliotis midae*	South Africa	[380]
abalone	*H. rubra*	Australia	[381]

## Data Availability

Not applicable.
